# How groundwater temperature is affected by climate change: A systematic review

**DOI:** 10.1016/j.heliyon.2024.e27762

**Published:** 2024-03-16

**Authors:** Elena Egidio, Domenico Antonio De Luca, Manuela Lasagna

**Affiliations:** a University of Torino, Earth Sciences Department, Via Valperga Caluso 35, 10125, Torino, Italy

**Keywords:** Groundwater temperature, Aquifer, Effects of climate change, Bibliographic review

## Abstract

Groundwater (GW) is sensitive to climate change (CC), and the effects have become progressively more evident in recent years. Many studies have examined the effects of CC on GW quantity. Still, there is growing interest in assessing the qualitative impacts of CC, especially on GW temperature (GWT), and the consequences of these impacts.

This study aimed to systematically review recently published papers on CC and GWT, determine the impacts of CC on GWT, and highlight the possible consequences.

The Scopus and Web of Science databases were consulted, from which 144 papers were obtained. After an initial screening for duplicate papers, a second screening based on the titles and abstracts, and following an analysis of topic applicability to this subject after examining the full text, 44 studies were included in this review. The analysed scientific literature, published in 29 different journals, covered all five continents from 1995 to 2023.

This review indicated that the subject of GWT variations due to CC is of global interest and has attracted significant attention, especially over the past two decades, with many studies adopting a multidisciplinary approach. A general increase in GWT was noted as a primary effect of CC (especially in urban areas); furthermore, the implications of this temperature increase for contaminants and GW-dependent ecosystems were analysed, and various applications for this increase (e.g. geothermal) were evaluated.

This review highlights that GWT is vulnerable to CC and that the consequences can be serious and worthy of further investigation.

## Highlights

• "Study reviewed 44 papers on climate change & groundwater temperature highlighting global interest".
• "Climate change impacts on groundwater temperature are discussed in 44 papers".
• "Groundwater-dependent ecosystems are affected by increasing groundwater temperature".
• "Groundwater temperature increase could be exploitable for geothermal & industrial purposes".
• "Consequences of groundwater temperature change due to climate change warrant further investigation".

## Graphical abstract

**Figure:**
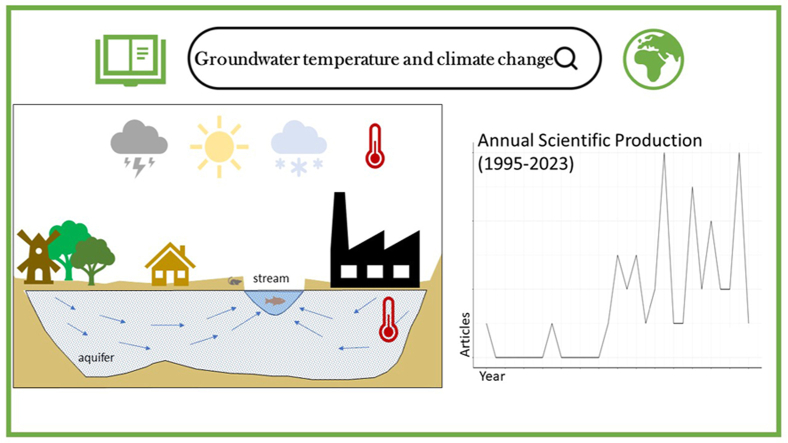

## 1 Introduction

In 1992, the First Assessment Report (FAR) of the IPCC [1] highlighted climate change (CC) as a global challenge with significant consequences. While the effects of CC are becoming increasingly visible, there are also “invisible” effects, particularly on groundwater (GW). Groundwater, the “invisible resource”, is sensitive to CC, and the effects have become progressively more evident in recent years. In the first IPCC Assessment Report [1], the word “groundwater” appeared only once, while in the latest IPCC Assessment Report [2], it was repeated 570 times, demonstrating a greater focus on this resource.

In 2022, World Water Day was dedicated to groundwater, emphasising the need to "make the invisible visible". As a result of this initiative, a UNESCO Report on Groundwater [3] was published that emphasised the pivotal role of GW in addressing poverty, ensuring access to food and water, generating quality employment opportunities, fostering socio-economic progress, and enhancing the ability of societies and economies to withstand the impacts of CC. Moreover, a study published in early 2023 [4] summarised key messages from various studies during 2022 related to GW and CC, emphasising the role of hydrogeologists in working toward the water-related Sustainable Development Goals (SDGs) of the United Nations [5] and creating innovative hydrogeological approaches to address GW-related issues. Scientific research on this topic over the past 30 years has mostly focused on the effects of CC on the quantitative depletion of GW resources (e.g. Refs. [[6], [7], [8], [9], [10], [11], [12], [13]]). Only in the last two decades has the topic of CC effects on GW also been analysed from a qualitative point of view (e.g. Refs. [14,15]).

One of the main effects of CC on GW is related to variations in water temperature, which are observed globally (e.g. Ref. [16]). However, given the delayed interest in the subject, long GWT time series are difficult to find in the literature. Where available, they are very short compared to time series data on air temperature (AT), making the study of GWT changes over time challenging (e.g. Refs. [17,18]). Other effects of groundwater temperature (GWT) variations are also evident, particularly about GW-dependent ecosystems, which have been studied by various authors (e.g. Refs. [19,20]). In fact, in these ecosystems, the quality of the GW and its temperature are fundamental to the proper balance and functioning of the habitat for all species within it. In addition, other effects related to GWT can be found in cold areas where permafrost is prevalent. Many authors over the years have focused on these cold areas by analysing the effects of CC on permafrost (e.g. Refs. [21,22]) and highlighting the progressive thawing and subsequent reduction in permafrost. Looking at the causes of this thawing, some studies have highlighted the fundamental role of GW in the active layer below the permafrost [[23], [24], [25], [26], [27], [28]], which also influences the thermal properties of permafrost [29]. In addition, it is also crucial to emphasise the negative aspects that result from permafrost thawing, such as the vulnerability of infrastructure built on frozen ground and the change in the thermal properties of permafrost due to this infrastructure [23,25,30,31]. Furthermore, permafrost thawing could lead to watercourses contamination by natural heavy metals [32,33]. Thus, understanding the physical processes that lead to permafrost thaw in the context of current CC is also crucial because this thawing can lead to variations in the hydrological cycle [26]. If the glaciers continue to shrink in the future, the water resources that feed the rivers and GW in cold areas could substantially decline [24]. Thus, the role of permafrost in mountain areas in the hydrological cycle would become crucial [34]. Another aspect related to GWT variations is addressed in the medical scientific literature (e.g. Ref. [35]), as temperature variations in GW can affect the quality of the water used for human purposes. In fact, several studies confirming the temperature dependency of viruses have been conducted. An infectious virus can survive in low-temperature GW for years [36]. Therefore, an increase in GWT could be positive in these cases since many viruses may become inactive at high temperatures, especially above 20 °C [37].

The opposite behaviour, however, is observed with regard to bacteria, as an increase in GWT leads to greater proliferation of bacteria, demonstrating how temperature is a key aspect in increasing bacterial diversity [38,39]. Nevertheless, a study carried out at piezometers used for monitoring a shallow aquifer exploited for geothermal energy showed a reduction in bacteria in the heat plume, suggesting that the thermal shock inside the heat exchangers can lead to fewer bacteria in the water [40]. Furthermore, it has been observed that an increase in GWT may also lead to changes in the drinking water quality (e.g. pH, O_2_, Mn), which could result in higher depuration costs [41].

The aim of this study is to conduct a systematic review of published papers to determine the impacts of CC on GWT and the resulting consequences, emphasising the increasing importance of studying the effects of CC on GWT and the need to understand its implications for GW resources and their related ecosystems.

## 2 Methods

### 2.1 Search strategy and inclusion criteria

The Scopus and Web of Science databases were consulted on February 8, 2023. The search queries used in both databases are as follows:

“TITLE (groundwater AND temperature AND climate)OR TITLE (aquifer AND temperature AND climate) OR AUTHKEY (groundwater AND temperature AND climate) OR AUTHKEY (aquifer AND temperature AND climate)“.

TITLE is the code used for searching words within the title of a document and AUTHKEY is the code used to search the keywords that the author(s) assigned to the document.

No type of linguistic restriction was applied in this database search. However, only papers written in English were considered in the full-text review. Abstracts, long abstracts or papers in languages other than English were omitted from the list.

### 2.2 Study selection

Covidence software [42] was used to facilitate screening the papers, allowing all authors to participate in the final decisions. The selection of studies was carried out according to the following procedure.

1. Importing of all the bibliographic records extracted from Scopus and Web of Science into the software;
2. Elimination of duplicate papers found in both databases;
3. The first selection phase was based on the titles and abstracts of the papers to exclude articles that were not related to the research topic;
4. Examination of the full text of the articles for a more thorough check of their content;
5. Final decision and discussion among the authors to select the final articles for consideration in this study;
6. Creation of a PRISMA [43] flow diagram to better visualise the selection.

Finally, two programs (Bibliometrix [44] and VosViewer [45]) were used to visualise the results of the bibliometric research better. Bibliometrix [44] is an R-tool that allows a BibText file to be imported, which in this case was derived from the merged results of the WOS and Scopus databases; this tool provides graphical representations to help with bibliometric data interpretation.

VosViewer [45] is a program that helps to visualise the links between the various keywords used in all the papers analysed.

During these paper analysis steps, one of the authors (E.E.) was tasked with the detailed analysis of all the articles found in the literature search. In cases of uncertainties, the third author (M.L.) reviewed the papers. In cases where these two authors disagreed, the second author (D.A.D.) was also consulted for a third point of view.

## 3 Results

### 3.1 Search results, study characteristics and data quality

The initial search of the Scopus and Web of Science databases yielded 144 articles (78 from Scopus and 66 from WOS). By eliminating duplicates, 80 articles were ultimately identified.

After reading the titles and abstracts, it was determined that all 80 articles met the selection criteria (English and full text) of the review. After reading the full text, 36 papers were deleted from the list because they were outside of this review's focus. At the same time, 44 studies were considered relevant to the study topic and were retained in this review.

**Fig. 1:**
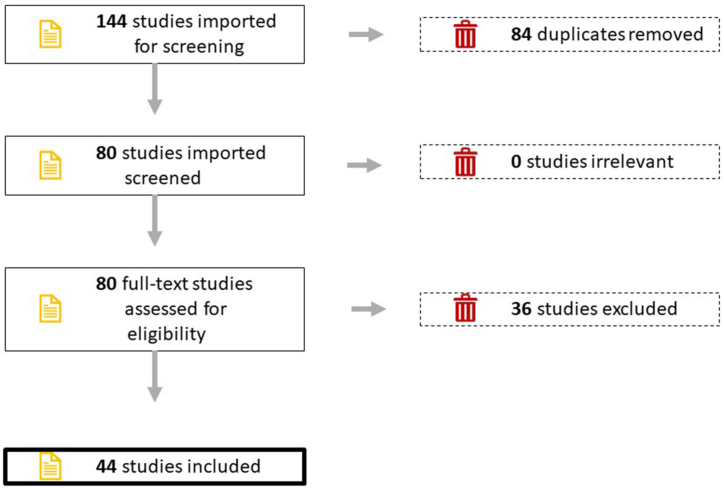
PRISMA flow diagram resulting from the bibliographic search performed on the documents found in the two analysed databases (Scopus and Web of Science).

The results of this process are depicted in a PRISMA [43] flow diagram (Fig. 1).

The list of the 44 analysed papers is shown in Table 1.

**Table 1:** List of all articles considered in the study, in alphabetical order according to first author, all articles considered for the search (authors, year of publication, title, journal of publication).

Authors	Publication Year	Title	Source
Andrushchyshyn, OP; Wilson, KP; Williams, DD [46]	2009	**Climate change-predicted shifts in the temperature regime of shallow groundwater produce rapid responses in ciliate communities**	**Global Change Biology**
Bastiancich, L; Lasagna, M; Mancini, S; Falco, M; De Luca, DA [47]	2022	**Temperature and discharge variations in natural mineral water springs due to climate variability: a case study in the Piedmont Alps (NW Italy)**	**Environmental Geochemistry and Health**
Benz, SA; Bayer, P; Blum, P [16]	2017	**Global patterns of shallow groundwater temperatures**	**Environmental Research Letters**
Blanco-Coronas, AM; Calvache, ML; Lopez-Chicano, M; Martin-Montanes, C; Jimenez-Sanchez, J; Duque, C [48]	2022	**Salinity and Temperature Variations near the Freshwater-Saltwater Interface in Coastal Aquifers Induced by Ocean Tides and Changes in Recharge**	**Water**
Burns, E.R.; Zhu, Y.; Zhan, H.; Manga, M.; Williams, C.F.; Ingebritsen, S.E.; Dunham, J.B [49].	2017	**Thermal effect of climate change on groundwater-fed ecosystems**	**Water Resources Research**
Carlson, AK; Taylor, WW; Infante, DM [50]	2019	**Developing precipitation- and groundwater-corrected stream temperature models to improve brook charr management amid climate change**	**Hydrobiologia**
Cavelan, A; Golfier, F; Colombano, S; Davarzani, H; Deparis, J; Faure, P [51]	2022	**A critical review of the influence of groundwater level fluctuations and temperature on LNAPL contaminations in the context of climate change**	**Science of the Total Environment**
Chu, C; Jones, NE; Mandrak, NE; Piggott, AR; Minns, CK [52]	2008	**The influence of air temperature, groundwater discharge, and climate change on the thermal diversity of stream fishes in southern Ontario watersheds**	**Canadian Journal of Fisheries and aquatic sciences**
Cogswell, C; Heiss, JW [53]	2021	**Climate and Seasonal Temperature Controls on Biogeochemical Transformations in Unconfined Coastal Aquifers**	**Journal of Geophysical Research - Biogeosciences**
Colombani, N; Giambastiani, BMS; Mastrocicco, M [54]	2016	**Use of shallow groundwater temperature profiles to infer climate and land use change: interpretation and measurement challenges**	**Hydrological Processes**
Dittbrenner, BJ; Schilling, JW; Torgersen, CE; Lawler, JJ [55]	2022	**Relocated beaver can increase water storage and decrease stream temperature in headwater streams**	**Ecosphere**
Egidio, E; Mancini, S; De Luca, DA; Lasagna, M [17]	2022	**The Impact of Climate Change on Groundwater Temperature of the Piedmont Po Plain (NW Italy)**	**Water**
Epting, J; Huggenberger, P [56]	2013	**Unraveling the heat island effect observed in urban groundwater bodies - Definition of a potential natural state**	**Journal of Hydrology**
Epting, J; Michel, A; Affolter, A; Huggenberger, P [57]	2021	**Climate change effects on groundwater recharge and temperatures in Swiss alluvial aquifers**	**Journal of Hydrology**
Fennell, J; Geris, J; Wilkinson, ME; Daalmans, R; Soulsby, C [58]	2020	**Lessons from the 2018 drought for management of local water supplies in upland areas: A tracer-based assessment**	**Hydrological Processes**
Gross-Wittke, A; Gunkel, G; Hoffmann, A [59]	2010	**Temperature effects on bank filtration: redox conditions and physical-chemical parameters of pore water at Lake Tegel, Berlin, Germany**	**Journal of Water and Climate Change**
Gunawardhana, LN; Kazama, S [60]	2009	**A use of global climate model output for site-specific assessment of climate change impacts on groundwater temperature**	**Symposium HS.2 at the Joint Convention of the International Association of Hydrological Sciences, IAHS and the International Association of Hydrogeologists, IAH 2009**
Gunawardhana, LN; Kazama, S [61]	2012	**Statistical and numerical analyses of the influence of climate variability on aquifer water levels and groundwater temperatures: The impacts of climate change on aquifer thermal regimes**	**Global and Planetary Change**
Gunawardhana, LN; Kazama, S [62]	2011	**Climate change impacts on groundwater temperature change in the Sendai plain, Japan**	**Hydrological Processes**
Gunawardhana, LN; Kazama, S; Kawagoe, S [63]	2011	**Impact of Urbanization and Climate Change on Aquifer Thermal Regimes**	**Water Resources Management**
Hemmerle, H; Bayer, P [64]	2020	**Climate Change Yields Groundwater Warming in Bavaria, Germany**	**Frontiers in Earth Science**
Horsak, M; Polaskova, V; Zhai, M; Bojkova, J; Syrovatka, V; Sorfova, V; Schenkova, J; Polasek, M; Peterka, T; Hajek, M [65]	2018	**Spring-fen habitat islands in a warming climate: Partitioning the effects of mesoclimate air and water temperature on aquatic and terrestrial biota**	**Science of the Total Environment**
Irvine, DJ; Kurylyk, BL; Cartwright, I; Bonham, M; Post, VEA; Banks, EW; Simmons, CT [66]	2017	**Groundwater flow estimation using temperature-depth profiles in a complex environment and a changing climate**	**Science of the Total Environment**
Johnson, Z.C.; Snyder, C.D.; Hitt, N.P [67].	2017	**Landform features and seasonal precipitation predict shallow groundwater influence on temperature in headwater streams**	**Water Resources Research**
Kaandorp, VP; Doornenbal, PJ; Kooi, H; Broers, HP; de Louw, PGB [68]	2019	**Temperature buffering by groundwater in ecologically valuable lowland streams under current and future climate conditions**	**Journal of Hydrology**
Kanno, Y; Vokoun, JC; Letcher, BH [69]	2014	**Paired stream-air temperature measurements reveal fine-scale thermal heterogeneity within headwater brook trout stream networks**	**Rivers Research and Applications**
Kurylyk, BL; Bourque, CPA; MacQuarrie, KTB [70]	2013	**Potential surface temperature and shallow groundwater temperature response to climate change: an example from a small forested catchment in east-central New Brunswick (Canada)**	**Hydrology and Earth System Sciences**
Kurylyk, BL; MacQuarrie, KTB [71]	2014	**A new analytical solution for assessing climate change impacts on subsurface temperature**	**Hydrological Processes**
Kurylyk, BL; MacQuarrie, KTB; Caissie, D; McKenzie, JM [72]	2015	**Shallow groundwater thermal sensitivity to climate change and land cover disturbances: derivation of analytical expressions and implications for stream temperature modeling**	**Hydrology and Earth System Sciences**
Kurylyk, BL; MacQuarrie, KTB; McKenzie, JM [73]	2014	**Climate change impacts on groundwater and soil temperatures in cold and temperate regions: Implications, mathematical theory, and emerging simulation tools**	**Earth-Science Reviews**
Kurylyk, BL; MacQuarrie, KTB; Voss, CI [21]	2014	**Climate change impacts on the temperature and magnitude of groundwater discharge from shallow, unconfined aquifers**	**Water Resources Research**
Leach, J.A.; Moore, R.D [74].	2019	**Empirical Stream Thermal Sensitivities May Underestimate Stream Temperature Response to Climate Warming**	**Water Resources Research**
Lee, B; Hamm, SY; Jang, S; Cheong, JY; Kim, GB [75]	2014	**Relationship between groundwater and climate change in South Korea**	**Geosciences Journal**
Mastrocicco, M; Busico, G; Colombani, N [76]	2019	**Deciphering Interannual Temperature Variations in Springs of the Campania Region (Italy)**	**Water**
Menberg, K; Blum, P; Kurylyk, BL; Bayer, P [77]	2014	**Observed groundwater temperature response to recent climate change**	**Hydrology and Earth System Sciences**
Morsy, K.M.; Alenezi, A.; Alrukaibi, D.S [78].	2017	**Groundwater and dependent ecosystems: Revealing the impacts of climate change**	**International Journal of Applied Engineering Research**
Noethen, M; Hemmerle, H; Bayer, P [79]	2023	**Sources, intensities, and implications of subsurface warming in times of climate change**	**Critical Reviews in Environmental Science and Technology**
Park, YC; Jo, YJ; Lee, JY [80]	2011	**Trends of groundwater data from the Korean National Groundwater Monitoring Stations: indication of any change?**	**Geosciences Journal**
Risley, JC; Constantz, J; Essaid, H; Rounds, S [81]	2010	**Effects of upstream dams versus groundwater pumping on stream temperature under varying climate conditions**	**Water Resources Research**
Rivers-Moore, NA; Dallas, HF [82]	2022	**A spatial freshwater thermal resilience landscape for informing conservation planning and climate change adaptation strategies**	**Aquatic conservation-marine and freshwater ecosystems**
Scheihing, K; Troger, U [83]	2018	**Local climate change induced by groundwater overexploitation in a high Andean arid watershed, Laguna Lagunillas basin, northern Chile**	**Hydrogeology Journal**
Sinokrot, B.A; Stefan, H.G; McCormick, J.H; Eaton J.G [84].	1995	**Modeling of climate change effects on stream temperatures and fish habitats below dams and near groundwater inputs**	**Climatic Change**
Taniguchi, M [85]	2002	**Estimations of the past groundwater recharge rate from deep borehole temperature data**	**Catena**
Taylor, CA; Stefan, HG [10]	2009	**Shallow groundwater temperature response to climate change and urbanization**	**Journal of Hydrology**

**Fig. 2:**
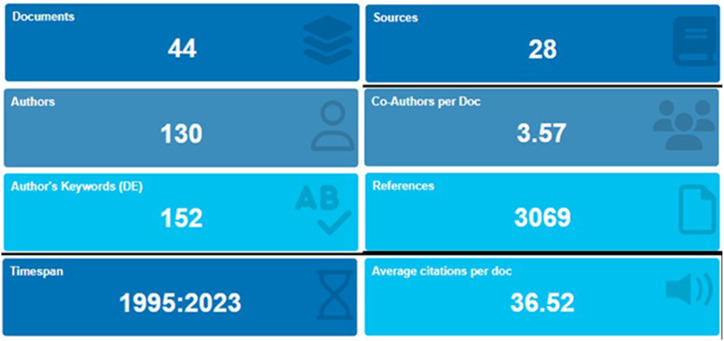
Summary of the key generic results of the bibliometric research carried out with the Bibliometrix software. (Documents = number of final documents considered for the review; Sources = number of journals from which the documents originated; Authors = total number of authors who contributed to all the articles analysed; Timespan: time period in which the articles were published; Co-authors per Doc: average number of co-authors who participated in the writing of each article; Author's Keywords: total number of keywords chosen by the author(s); References: total number of bibliographic citations of all the documents; Average Citations per Doc: average number of times each article was cited).

In addition, the generic bibliometric search results from the use of Bibliometrix [44] software are shown in Fig. 2.

**Fig. 3:**
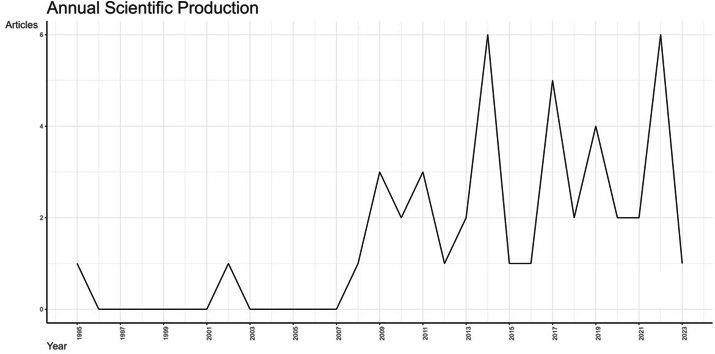
Number of articles published each year (in the time interval of 1995–2023).

**Fig. 4:**
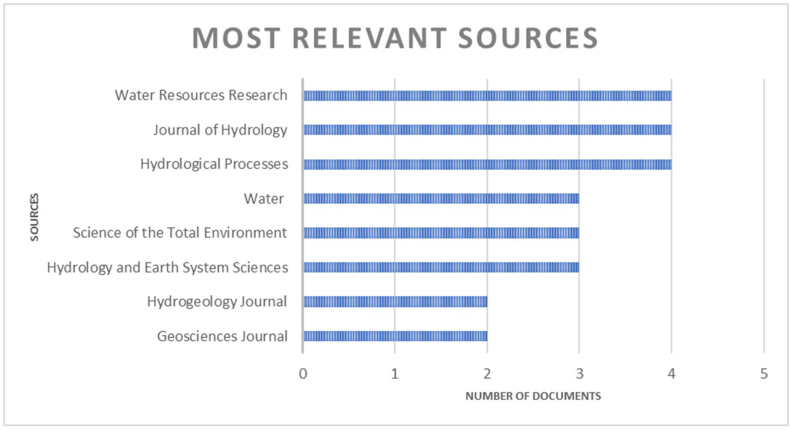
Number of papers published in each journal (only journals with at least two of the analysed articles are presented).

The analysed scientific research (a total of 44 papers after selection) covers a period from 1995 to 2023, with an increase in papers from 2008 onwards (Fig. 3). The analysed papers were published in twenty-eight different indexed journals (Fig. 4).

**Fig. 5:**
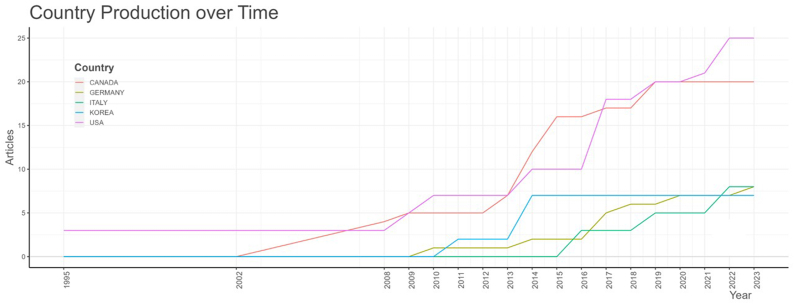
Number of articles published per country (the scientific affiliations of all of the authors of each article are considered. Therefore, each article is included multiple times if it has multiple co-authors; e.g.: a paper with 3 authors would be included 3 times). The five countries with the highest number of affiliated authors are shown in the figure.

In the detailed analysis based on the affiliation of all the authors (130 authors in total), it was observed that the country with most publications was the United States, followed by Canada, Italy and Germany (Fig. 5).

**Fig. 6:**
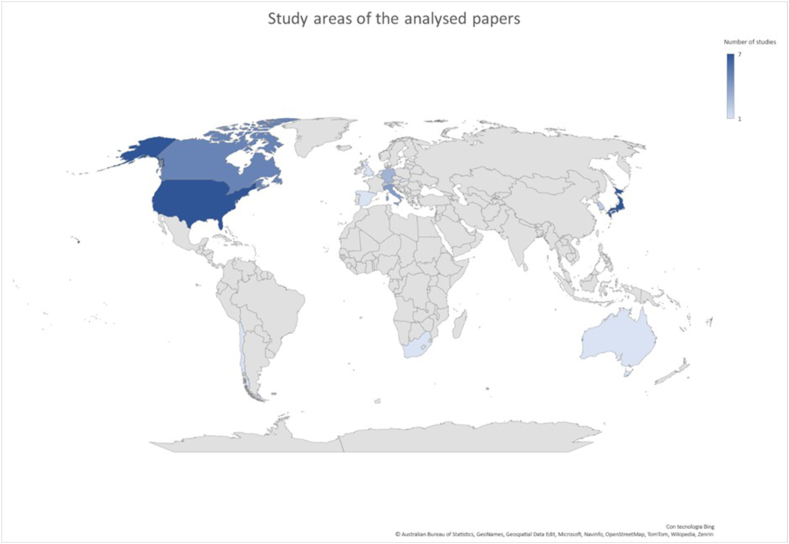
Geographical location of the study areas considered by the articles. More intense colours indicate a greater number of articles focusing on a particular area. (In this graphical representation, articles with a global point of view have been excluded). (For interpretation of the references to colour in this figure legend, the reader is referred to the Web version of this article.)

Moreover, the bibliometric research indicated that relevant studied were carried out in all continents (Fig. 6) and particularly.

• Europe, 13
• North America, 12
• Asia, 9
• South America, 1
• Africa, 1
• Oceania, 1

In addition, 7 of the analysed articles (16% of the total) had a global perspective.

**Fig. 7:**
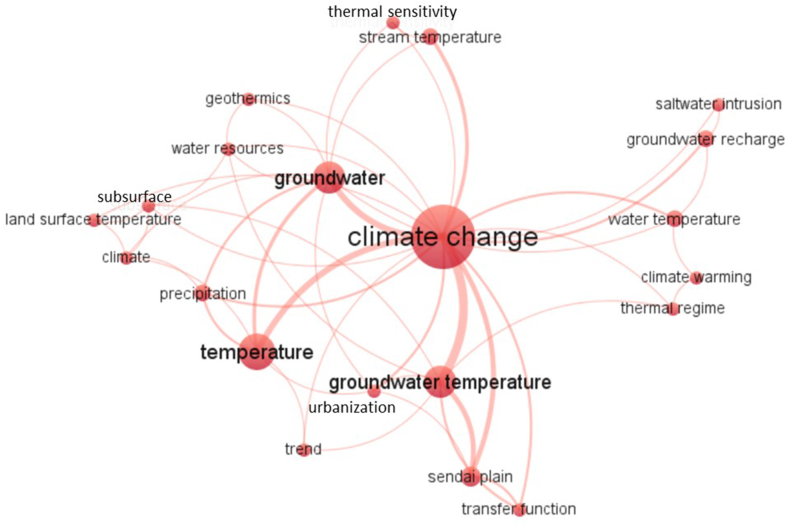
Graphic representation resulting from the use of the VosViewer software in which the relationships between the various Author Keywords used in the analysed articles are highlighted. The lines connect words that were used together in at least one document, and the size of the dots for each word is indicative of the number of documents in which the word was used.

Next, the Author's Keywords most frequently used in the selected articles were analysed by creating a map with VosViewer [45]. Out of a total of 152 author keywords, those that were repeated at least 2 times were selected, which totalled 21 keywords (Fig. 7). The keywords that appeared most frequently were very similar to 3 of the 4 words that were used in the database search (GW, temperature and climate).

### 3.2 Primary outcome

All of the analysed articles noted that GW is affected by CC. CC has various impacts, and among these, all authors point out changes in GWT.

Analysing all the texts of the 44 papers, it was possible to identify three main thematic categories listed below in order of frequency.

• General hydrogeology: 24 articles
• Groundwater ecosystems: 17 articles
• Industrial applications and energy: 3 articles

Where two or more themes were present in an article, it has been chosen to categorise it under the prevalent theme. For ease of reference, the articles for each topic are summarised in Table 2.

**Table 2:** Table in which the articles are divided according to the three subject categories identified.

GENERAL HYDROGEOLOGY		GROUNDWATER ECOSYSTEMS	INDUSTRIAL APPLICATIONS AND ENERGY
Bastiancich et al., 2021 [47]	Menberg 2014 [77]	Andrushchyshyn et al., 2009 [46]	Epting and Huggenberger 2013 [56]
Benz et al., 2017 [16]	Noethen et al., 2023 [79]	Burns et al., 2017 [49]	Fennell et al., 2020 [58]
Blanco-Coronaset al., 2022 [48]	Park et al., 2011 [80]	Carlson et al., 2019 [50]	Hemmerle and Bayer 2020 [64]
Cavelan et al., 2022 [51]	Risley et al., 2010 [81]	Chu et al., 2008[ 28]	
Colombani et al., 2016 [54]	Scheihing and Tröger 2018 [83]	Dittbrenner et al., 2022 [55]	
Cogswell and Heiss 2021 [53]	Taniguchi 2002 [85]	Gross-Wittke et al., 2010 [59]	
Egidio et al., 2022 [17]	Taylor and Stefan 2009 [10]	Horsák et al., 2018 [65]	
Epting et al., 2021 [57]		Johnson et al., 2017) [67]	
Gunawardhana and Kazama 2009 [60]		Kaandorp et al., 2019 [68]	
Gunawardhana et al., 2011 [63]		Kanno et al., 2014 [69]	
Gunawardhana and Kazama 2012 [61]		Kurylyk et al., 2013 [70]	
Gunawardhana and Kazama 2011 [62]		Kurylyk et al., 2014 [73]	
Irvine et al., 2017 [66]		Kurylyk et al., 2014 [21]	
Kurylyk and MacQuarrie 2014 [71]		Leach and Moore 2019 [74]	
Kurylyk et al., 2015 [72]		Morsy et al., 2017 [78]	
Lee et al., 2014 [75]		Rivers-Moore 2022 [82]	
Mastrocicco et al., 2019 [76]		Sinokrot 1995 [84]	

### 3.3 Secondary outcome

#### 3.3.1 General hydrogeology

As far as general hydrogeology studies are concerned, all the studies considered here showed an influence of CC on GWT. More specifically, many authors worldwide have focused their studies on the relationship between AT and GWT.

In Japan (Sendai Plain) Gunawardhana and Kazama [60] estimated that GWT will increase between 1.1 and 2.6 °C from 2060 to 2099 due to increases in AT (+3.3 °C) and precipitation. Park et al. [80] observed that between 1996 and 2008, GWT in South Korea showed increasing trends with average increases of approximately 0.04–0.09 °C/yr due to the effects of global warming, and they expect this value to increase in upcoming years. Additionally, Lee et al. [75] highlighted an increasing GWT trend in South Korea from 2000 to 2010 due to CC, with a slope of 0.1006 °C/yr (more pronounced in the winter period).

Other authors, such as Egidio et al. [17] in Italy and Menberg et al. [77] in Germany, focused on the comparison of GWT and AT variations, both observing a more marked increase in AT, and concluding that aquifers, especially shallow aquifers, are affected by CC and are therefore vulnerable. However, GWT is more resilient than AT to CC. Moreover, the thermal signals in AT are attenuated and lag at the subsurface, causing a more gradual increase in GWT. Other authors have studied GWT variations in coastal aquifers. Blanco-Coronas et al. [48] examined temperature distribution in coastal aquifers. Periodic thermal oscillations are influenced by recharge and tides, with a greater impact in deeper areas. The oscillations are related to the movement of the freshwater-saltwater interface and the upward flow from the aquifer. Temperature differences are amplified between layers with different hydraulic conductivity. Colombani et al. [54] compared three different measurement techniques of vertical GWT profiles in an Italian coastal aquifer to identify the most robust acquisition techniques. Accurate temperature proﬁles were crucial for inferring the inﬂuence of land use and CC on GW. Cogswell and Heiss [53] studied the roles of global and seasonal variability of GW and coastal ocean temperatures on the spatial and temporal trends of the N cycle in coastal aquifers. More specifically, they noted that GWT has a strong control on the reactivity of coastal aquifers: e.g. nitrate removal rates vary from 5% to 88% for different thermal regimes globally, which has serious implications in a warming environment.To better observe the effect of CC on GW in the absence of anthropogenic effects, some authors analysed springs in mountainous areas. Bastiancich et al. [47], in northern Italy, highlighted that approximately one-third of the analysed springs in their study showed a GWT increase (+0.007 °C/yr) associated with the increase in AT (up to +0.03 °C/year) over the period 2001–2018. In addition, cross-correlation tests demonstrated a close relationship between AT and GWT, with a time-lag of between 0 and 3 months, and between spring flow and AT, with a time-lag of between 1 and 3 months. Mastrocicco et al. [76], in southern Italy, also observed an increase in GWT of approximately +2.0 °C over the period from 2002 to 2017. This was well associated with rising minimum atmospheric temperatures, but not with rising mean and maximum temperatures. To better analyse the effect of land use, some authors have focused their investigations on GWT increases in urban environments. Epting et al. [57] analysed Swiss alluvial aquifers and observed that in urban areas, human influences (e.g. increased thermal utilisation of the subsoil and heat waste from underground structures) are likely to dominate compared to CC effects. For example, measurements from the city of Basel showed that the GWT increased by an average of 3.0 ± 0.7 °C over the period from 1993 to 2016. Furthermore, they observed that GWT was strongly affected by changes in recharge patterns, especially in shallow aquifers with thin GW-saturated zones; in contrast, changes in GWT were strongly mitigated and could only be predicted over long periods within deep aquifers with thick GW-saturated zones. In addition, they hypothesised that increased GW recharge during high runoff could strongly influence the GWT and that seasonal changes in GW recharge processes could influence future GWT. Gunawardhana et al. [63], in Japan, also observed that in urban areas, 75% of the increase in GWT (0.7–1.0 °C) was related to urbanisation effects over the past 60 years. Moreover, using a forecasting approach, they estimated an aquifer temperature change of 1.8–3.7 °C by 2080 due to CC, which was significantly higher than the effect of urbanisation on the aquifer temperature. In a study on the effects of urbanisation and CC in northern temperate climate areas, Taylor and Stefan [10] observed that seasonal temperature cycles penetrated the soil at depths of approximately 10–15 m. Moreover, they estimated that a fully industrialised city centre at the latitude of Minneapolis could have a nearly 3 °C warmer GWT than a rural area under the same climatic conditions (4 °C in the CO_2_ doubling scenario). Noethen et al. [79], recognising the importance of temperature variations for GW quality, proposed a classification of underground heat sources based on the geometry, the scale at which the ground is warmed, the process that produces the heat and the intention to discharge the heat. This could better assess the impact on GW and the GW-dependent ecosystems. In a global study, Benz et al. [16] attempted to approximate shallow GWT using satellite-derived land surface temperatures. They highlighted the presence of a global bias between shallow GWT and Earth surface temperatures, mainly due to evapotranspiration and snow cover. Therefore, considering only these two processes, the global GWT at shallow depths was assessed with a resolution of approximately 1 km.

Several authors have attempted to observe the various effects that CC can have on GWT and GW quality. Cavelan et al. [51] discussed the effects of CC on Light Non-Aqueous Phase Liquid (LNAPL) sites and observed that higher temperatures and a greater amplitude of GW table variations probably increased the rate of biodegradation, LNAPL mobility and diffusion through the smear zone, favouring the liberation of LNAPL compounds into both the atmosphere and the aquifer, but reducing their mass and longevity. Risley et al. [81] observed that an increase in GW withdrawals led, in rivers, to water warming in summer and water cooling in winter highlighting the importance of GW input in rivers that allow thermal equilibrium within the river. In the Chilean Andes, Scheihing and Tröger [83] observed how a severe aquifer overexploitation can induce local CC. More specifically, they highlighted how GW is critical for maintaining thermal equilibrium, observing that lowering the water table to 2 m below ground level (bgl) caused warming of the bare ground surface influenced the lower atmosphere and reduced thermal exchange between the aquifer (constant GWT ∼10 °C) and the lower atmosphere at night, resulting in a sharp decline in the minimum AT. They also noted that the critical water level lowering threshold was 2–3 m bgl.

Other authors have highlighted the importance of knowing the variation of GWT with aquifer depth for estimating the recharge rate (Gunawardhana and Kazama [61,62] and Irvine et al. [66]) and surface temperature history (Taniguchi [85]).

The role of GWT as an important water quality parameter is largely recognised, and many authors have tried to analyse the response of GWT to atmospheric CC by implementing new analytical solutions. Kurylyk and MacQuarrie [71] proposed a new analytical solution derived from a 1-D flow model verified by numerical methods using the finite element SUTRA code. Their simulation results indicated that shallow GW warming can be accelerated by recharge and that the upwards of GW can increase the heating of the subsoil with depth. The same authors (Kurylyk et al. [72]) also proposed the application of several analytical solutions to predict the time and magnitude of GWT changes due to seasonal and long-term variations in the Earth's surface temperature. In particular, warming was found to depend on the surface warming rate, subsurface thermal properties and GW velocity. Finally, they emphasised the limits of using short-term air and water temperature recordings in future predictions.

#### 3.3.2 Groundwater ecosystems

GW ecosystems are strongly connected to GWT, and consequently many studies have stressed the importance of predicting the impact of CC on GWT. The effects of CC on freshwater ecosystems are substantial and have been the subject of much research worldwide.

Morsy et al. [78] reported the impacts of CC on GW-dependent ecosystems and recommended management options to adapt to these impacts. More specifically, changes in temperature and precipitation could have an unavoidable effect by altering the GW recharge and jeopardising the survival of biological communities through the interruption of key biological processes. Rivers-Moore [82] pointed out the importance of identifying the thermal features of rivers and defining the relative ecosystem resilience to evaluate the potential consequences of CC on aquatic ecosystems. Freshwater thermal resilience was affected by different parameters, including connections to GW parameters, such as the water table depth. Kurylyk et al. [73] considered the impacts of future CC on subsurface thermal regimes in cold and temperate regions, highlighting that subsurface thermal environments in colder regions are highly sensitive to CC. In particular, in regions with permafrost, variations in soil temperature could cause changes in surface and subsurface hydrological patterns and alter ecosystems that rely on cold water runoff or discharge.

Other authors have focused on the implementation of numerical models to explain better the effects of CC on GWT and GW-dependent ecosystems. Sinokrot et al. [84] developed a deterministic heat transport model to evaluate the effects of CC on stream temperature and fish habitats below dams and GW sources. In particular, they simulated the loss of most available aquatic habitat under different CC scenarios. Moreover, they observed that the influence of CC on the stream temperatures beneath the dams was more severe when the water discharge came from the epilimnion (reservoir surface) rather than the hypolimnion (deep water) of the GW, making the GW-fed streams more resilient to CC. Additionally, Burns et al. [49] used the steady-state solutions of heat-transport equations to pinpoint processes controlling the long-term thermal response of springs to CC. Transient solutions were applied to assess the time needed for new heat signals to reach GW-dependent ecosystems. According to the authors, these estimates will help land managers and ecologists identify vulnerable GW-dependent ecosystems, develop environmental monitoring programmes, and plan adaptive modifications to available habitat. A linear modelling technique was proposed by Johnson et al. [67] to determine the effects of surface GW infiltration and AT on stream temperatures. They discussed the need for CC studies to consider GW-surface water dynamics when predicting future thermal thresholds for stream biota.

Understanding that changes in GWT could negatively impact riverine ecosystems, Kurylyk et al. [70] used projected surface temperature data in a CC scenario to show that shifts in GWT will have seasonal variability at shallow depths (1.5 m), but will be seasonally stable and roughly equal to the annual mean surface temperature change at greater depths (8.75 m). Moreover, in accordance with predicted future increases in GWT, they highlighted that the thermal susceptibility of baseflow-dominated streams to decadal CC may be higher than that indicated by previous research. The same authors (Kurylyk et al. [21]) studied CC inﬂuence on GWT and ﬂow rates, and their impacts on riverine ecosystems in a small, unconﬁned aquifer that experiences seasonal freezing and thawing. Their simulation indicated a possible increase in the flow rate (up to 34%) and temperature (up to 3.6 °C) of the GW discharge in the nearby river over the summer months due to AT and precipitation increases. This could create a critical situation for salmonids and other aquatic species during hot summer periods. Furthermore, the thermal responsivity of the GW was found to be highly reliant on the dimensions of the aquifer. Kanno et al. [69], in analysing CC impacts on stream ﬁsh distributions, suggested that regional stream temperature models are not optimal for completely reproducing the thermal variation at the local scale and may inaccurately represent the thermal resilience of stream networks. Moreover, GW appears to have a role in building the spatial thermal variation of streams at the ﬁeld scale, which can be characterised to assess the impacts of CC on GW species accurately. In a study conducted in the Netherlands, Kaandorp et al. [68] concluded that GW infiltration appears to buffer the effect of climate warming, potentially making GW-dominated watercourses more climatically resilient and suitable for aquatic biota that are very sensitive to changes in GWT. Consequently, the protection of GW resources from CC impacts is relevant for the viability of aquatic species in GW-fed systems because GW infiltration supports their flow and buffers temperature extremes.

Gross-Wittke et al. [59] applied an experimental setup on Lake Tegel, Germany, to simulate water heating and its effects on biological structure and ecosystem processes. Potential effects on self-purification processes were observed, which were explicitly due to changes in the stratification and circulation characteristics of the lake, and to the intensification of metabolic processes and biodegradation efficiency, which are responsible for water self-purification. They observed denitrification processes, such as the microbial-catalysed reduction of NO3 and the reduction of Mn4+ after oxygen depletion. However, no obvious effects of water heating on redox-chemical processes and microbial activity were detected. Horsák et al. [65] analysed the influence of mesoclimate air and local spring water temperature on the variation in the constitution of aquatic (macroinvertebrates), semi-terrestrial (plants) and terrestrial (land snails) spring pond biota. They observed that the influence of temperature was most significant for some species and that the importance of mesoclimatic temperature grew with terrestriality. Furthermore, the water temperature strongly influenced the aquatic and semi-aquatic biota of the springs. Finally, they noted that forecasting models based purely on AT can yield distorted estimates of future changes in spring peatland communities, as their aquatic and semi-terrestrial components are largely influenced by water temperature, which may be altered by local hydrological and landscape contexts.

Some authors have tried to better understand the effect of GWT variations on specific species that live in GW-dominated ecosystems. For example, many authors have analysed fish communities in relation to CC. Chu et al. [52] analysed the relationships between AT, GW discharge, and CC on the thermal diversity of stream fishes in southern Ontario watersheds. Indeed, the interaction between AT and GWT provides thermally diverse habitats in streams. They observed that the fishes and the thermal diversity of fish habitats were less sensitive to CC in watersheds with high levels of GW discharge. Leach and Moore [74] highlighted the relationship between AT and GWT and how it can possibly negatively impact cold-water fish such as salmon. Furthermore, according to the authors, GW-fed streams can only withstand warming in the short to medium term due to their delayed GWT response. For the long-term assessment of future temperature changes in streams is crucial for developing effective management responses for these habitats. Carlson et al. [50] focused on GW-dominated streams, emphasising how GWT increases or GW input reductions (due to reduced precipitation) would affect brook-char survival and growth in cold-water habitats. Andrushchyshyn et al. [46] focused on ciliates (unicellular organisms that live in many different ecosystems dominated by water). The observed that the distribution of these organisms was strongly correlated with GWT (highlighting how it was directly influenced by GWT but also indirectly affected by the level of dissolved oxygen, ammonia and nitrate levels and depth). They forecasted that the overall density of ciliates in spring GW ecosystems would increase in response to increasing temperatures, but species richness would decrease. Beavers also appeared to be a focal species in GW-dominated streams. In a study in Canada, Dittbrenner et al. [55]found that beavers could provide potential climate adaptation services in these environments. In fact, by moving beavers into GW-dominated streams, their consequential damming and pond forming habits increased the water retention, stream quenching and riparian ecosystem resilience of the streams within one year.

#### 3.3.3 Industrial applications and energy

Regarding industrial applications and energy, three papers on applications of GWT variations were identified.

One of the applications is geothermal energy. In a study conducted in the city of Basel (Switzerland), Epting and Huggenberger [56] observed a GWT increase of 9 °C over the natural state from 1970 to 2010. They examined the factors that influenced the urban GW regime and recommended better use of the higher geothermal potential in the urban area, since the GW (or at least most of it) has only been used for cooling purposes. The authors recommended foundations for a configuration of combined, heat-balanced heating and cooling systems. In a study conducted in Bavaria (Germany); Hemmerle and Bayer [64] showed that the annual thermal energy gained in GW bodies under 15 m due to CC was only one-third of the state's heat demand, which emphasised the geothermal potential linked to the variation of natural heat fluxes at the ground surface. Finally, in a study that aimed to analyse the effects of the 2018 Scottish (UK) drought, Fennel et al. [58] highlighted the consequences of increased GWT (in response to CC-related events) on industry, both in terms of water quantity and quality (temperature). The authors, focusing on the whisky distillery industry in the area, pointed out that GW is used for industrial cooling processes and that, therefore, an increase in its temperature might lead to changes and economic losses. The outcomes demonstrate the potential opportunities to preserve water volumes and temperatures through conscientious GW management: this would entail combining storage and infiltration measures to recharge the GW, by using nature-based solutions to improve resiliency to hydro-climatic extremes.

## 4 Discussion and conclusions

This bibliometric research shows that in all 44 analysed papers, the impact of CC on GWT is evident.

In particular, the authors who focused their studies on actual GWT variations carried out statistical analyses highlighting the relationships between GWT and AT variations. All papers showed an increase in GWT in connection with a CC-related increase in AT in different environments with greater evidence in urban areas. In addition, an increase in GWT also caused problems related to seawater intrusion in coastal areas. Moreover, many authors referred to water quality aspects in connection with an increase in GWT (e.g. nitrate and LNAPL).

The authors that delved into GW-dependent ecosystems all pointed out that an increase in GWT would lead to imbalances within these ecosystems that would be difficult to remedy. An increase in GWT affects the equilibria within these ecosystems, making them less suitable for living organisms that inhabit them. Furthermore, this research highlighted how the GWT also influences other environments, as GW is fundamental to the ecosystem balance of other habitats, such as rivers and lakes.

Finally, other authors have highlighted how GWT increases could be exploited for geothermal purposes and how modifications can impact industrial applications. A clear increase in the GWT, especially in urbanised areas, is observed and it was proposed by these authors as a method for industrial heating systems in urban areas. Furthermore, they emphasised that GW is often used for industrial processes and that changes in GWT could affect these processes.

From the analyses of the papers, it could be concluded that the subject of GWT variations is a multidisciplinary issue of global interest. However, it was evident from our bibliometric research that the Global South is underrepresented both in terms of authors and, above all, in terms of analysed areas. This is unfortunate, as the Global South is currently suffering the most from the effects of CC.

This bibliometric research did not include all of the papers related to this topic and that are present in the literature due to various factors (e.g. the database query may have been too specific, there may have been language problems, the search was limited to only two databases, etc.)

For example, it was not possible to investigate the effects of CC on GWT in cold permafrost-dominated areas, even though these effects are occurring even more rapidly in those areas than in southern regions, and this analysis is of extreme importance. This is an interesting and critical component that is required to achieve a full understanding of the issue at hand and certainly requires further study in the future.

Nevertheless, it is evident that GWT variations due to CC has been of paramount interest over the past two decades and that this interest continues to increase. This review observed a lack of homogeneity in the GWT methodology. Standardisation of data measurement methods is necessary to obtain comparable data on a regional (and subsequently global) scale. In fact, the GWT has been analysed using different instruments, at different depths, and over different measurement time intervals that are sometimes not comparable. For this reason, we recommend using continuous measuring instruments (with at least one measurement per week) to collect detailed data and construct good time series for adequate data interpretation. However, using instruments for continuous temperature measurement requires proper maintenance to avoid instrumental drift, which is common. Consequently, one calibration per year is recommended. Moreover, it is crucial to carefully assess and determine the appropriate probe placement within the water column. However, it isn't easy to select a standard depth as several variables influence the GWT, e.g., the thickness of the unsaturated zone, the depth of the water column over the probe, and the presence of a geothermal gradient. Hence, we recommend measuring at least two values whenever a GWT measurement campaign is conducted: the probe depth in relation to the water table and the depth of the water table with respect to the topographical surface. Another suggestion is to, where possible, take several measurements at different depths so that the temperature variations within the water column can be recorded.

Finally, the dedication of World Water Day to GW in 2022 was instrumental in emphasising the importance of GW resources in the context of global CC.

This review indicated that GW is vulnerable to CC, and needs to be monitored and protected because it is a fundamental resource in the context of the climate crisis. It is also evident from this review that through the study and monitoring of GW it is possible to observe effects of CC that would otherwise be invisible.

There is growing concern regarding the impacts of CC on GW, particularly on GWT. While the scientific community is starting to acknowledge this problem, there is a deficiency in communication strategies to convey this matter to the general public and various stakeholders, who are key players in the mitigation of CC. As a result, the effects of CC on GW, which are generally invisible, tend to be disregarded.

The global scientific community is responsible for raising public awareness of the problems related to GWT variations. Continuous investigation of this matter is critical and will enable improved management of global water resources within the context of CC.

## Data availability

Data will be made available on request.

## CRediT authorship contribution statement

**Elena Egidio:** Writing – review & editing, Writing – original draft, Visualization, Software, Methodology, Investigation, Formal analysis, Data curation, Conceptualization. **Domenico Antonio De Luca:** Supervision. **Manuela Lasagna:** Writing – review & editing, Writing – original draft, Visualization, Validation, Supervision, Methodology, Investigation, Funding acquisition, Data curation, Conceptualization.

## Declaration of competing interest

The authors declare that they have no known competing financial interests or personal relationships that could have appeared to influence the work reported in this paper.
